# How to Estimate Fat Mass in Overweight and Obese Subjects

**DOI:** 10.1155/2013/285680

**Published:** 2013-04-10

**Authors:** Lorenzo Maria Donini, Eleonora Poggiogalle, Valeria del Balzo, Carla Lubrano, Milena Faliva, Annalisa Opizzi, Simone Perna, Alessandro Pinto, Mariangela Rondanelli

**Affiliations:** ^1^Medical Physiopathology, Food Science and Endocrinology Section, Food Science and Human Nutrition Research Unit, Experimental Medicine Department, Sapienza University of Rome, 00185 Rome, Italy; ^2^Section of Human Nutrition and Dietetics, Endocrinology and Nutrition Unit, Department of Applied Health Sciences, Faculty of Medicine, University of Pavia, ASP, 27100 Pavia, Italy

## Abstract

*Background*. The prevalence of overweight and obesity is increasing and represents a primary health concern. Body composition evaluation is rarely performed in overweight/obese subjects, and the diagnosis is almost always achieved just considering body mass index (BMI). In fact, whereas BMI can be considered an important tool in epidemiological surveys, different papers stated the limitations of the use of BMI in single individuals. *Aim*. To assess the determinants of body composition in overweight and obese subjects. *Methods.* In 103 overweight or obese subjects (74 women, aged 41.5 ± 10 years, and 29 men, aged 43.8 ± 8 years), a multidimensional evaluation was performed including the assessment of body composition using Dual Energy X-Ray Absorptiometry (DXA), anthropometry, bioimpedance analysis (BIA), and biochemical parameters (total cholesterol, triacylglycerol, HDL- and LDL-cholesterol, free fatty acids and glycerol, glucose, insulin, C-reactive protein, plasma acylated and unacylated ghrelin, adiponectin, and leptin serum levels). *Results.* BMI does not represent the main predictor of FM estimated by DXA; FM from BIA and hip circumference showed a better association with FM from DXA. Moreover, models omitting BMI explained a greater part of variance. These data are confirmed by the predictive value analysis where BMI showed a performance similar to a “coin flip.”

## 1. Introduction

The prevalence of individuals who are classified as overweight or obese is increasing all over the world, representing a primary health concern due to the relationship between obesity and a number of diseases, disabilities, comorbidities, and mortality [1, 2].

The definition of obesity should consider not only the increase of body weight but more precisely the increase in body fat mass [3–5]. However, body composition evaluation is rarely performed in overweight and obese subjects, and the diagnosis is often achieved just considering body mass index (BMI = kg/m^2^). Even important Government Institutions suggest to use BMI to determine the presence of overweight and obesity [6].

The widespread use of BMI depends on its safety and minimal costs, and also on a rash and uncritical use of an epidemiological tool in clinical practice, conflicting with the advice of the inventor of BMI who first applied it to epidemiology. In 1972, the physiology professor and obesity researcher Ancel Keys published a landmark study encompassing more than 7,400 men in five countries [7]. Keys examined which of the height-weight formulas matched up best with each subject's directly measured fat mass (FM). It turned out that the best predictor was Quételet's index: body weight divided by height squared. Keys renamed this number as the *body mass index*. But BMI was explicitly cited by Keys as being appropriate for *population* studies and inappropriate for individual diagnosis. Nevertheless, due to its simplicity, it came to be widely used for individual diagnosis, despite its inappropriateness.

In fact, while BMI can be considered an important tool in epidemiological surveys, different papers stated the limitations of the use of BMI in single individuals [8–11] because of its incapacity to distinguish body components (fat mass and lean body mass in particular). 

On the contrary, it is pivotal to have a reliable estimation of FM both at the initial as well as at the outcome evaluation of obese subjects.

The purpose of this study is to verify the determinants of body composition in a population of overweight and obese subjects and to propose a different model of estimation of FM of these subjects when reliable equipments for the evaluation of body composition are not available.

## 2. Methods

### 2.1. Participants

This study was based on the baseline data from a randomised controlled trial aimed at the evaluation of the effects of 2-month consumption of a combination of bioactive food ingredients on changes in body composition, satiety control, thermogenesis, and serum markers of lipolysis [12]. 

The study was performed under the approval of the Ethics Committee of the Department of Internal Medicine and Medical Therapy at University of Pavia and registered at ClinicalTrials.gov (Clinical Trial Registration no. NCT01806493). The informed consent to the study was obtained by all the participants or their legal representatives. Healthy males and females aged from 25 to 45 years, with a BMI greater than 25 kg/m^2^ and less than 35 kg/m^2^, were eligible for the study. All subjects underwent physical examination, anthropometric assessment, and routine laboratory tests. The complete medical history was collected for all the subjects. Individuals who were pregnant or lactating or had any disease potentially affecting body composition and laboratory evaluation were excluded from the study; especially, severe hepatic or renal disease, unstable cardiovascular disease, uncontrolled hypertension, active cancer, and surgery for weight loss were the main exclusion criteria. 

### 2.2. Multidimensional Evaluation

After a 12-hour fasting, and abstinence from water since midnight, the subjects arrived at around 8:00 AM, using motorised transportation, at the Endocrinology and Clinical Nutrition Unit of Azienda di Servizi alla Persona di Pavia, University of Pavia (Italy) and at the Dietetic and Metabolic Unit, “Villa delle Querce” Clinical Rehabilitation Institute in Rome (Italy). 

Blood sampling for routine blood analysis and for the measurements of leptin, adiponectin, ghrelin, insulin, glycerol, and free fatty acid levels, as well as the assessment of body composition by dual energy X-ray absorptiometry (DXA) and anthropometry, was performed in the fasting state at baseline. 

#### 2.2.1. Body Composition Measurements

Body composition was measured using DXA (Lunar Prodigy DEXA, GE Medical Systems, Waukesha, WI). The *in vivo* coefficients of variation were 4.2% and 0.48% for fat and lean mass, respectively. Central fat, defined as the approximation of the visceral fat, was assessed with DXA, measuring the fat percentage corresponding to an ideal rectangle defined from the upper edge of the second lumbar vertebra to the lower edge of the fourth lumbar vertebra. The vertical sides of this area were the continuation of the lateral sides of the rib cage [13]. All measurements for each parameter were gathered by the same investigator.

*Anthropometry. *The following anthropometric measurements were performed in all subjects:

- body weight and height;
- biceps (BSF), triceps (TSF), suprailiac (SISF), and subscapular (SSSF) skinfold thicknesses;
- waist circumference (W), hip circumference (H), arm circumference (AC), and calf circumference (CC).
- In order to avoid the interassessor variability, anthropometric variables were measured by a unique investigator following a standardized technique [14]

Using the aforementioned anthropometric parameters, the following variables were calculated:

- body mass index (BMI): weight (kg)/height^2^ (m^2^);
- waist to hip ratio (WHR);
- arm muscle area (AMA) (cm^2^) = AC⁡−(*π*∗TSF)^2^/4*π*;
- arm fat area (AFA) (cm^2^) = (AC^2^/4*π*)–AMA;
- muscle arm circumference (MAC) (cm) = AC⁡−(*π*∗TSF).
 

*Bioimpedance Analysis (BIA).* Whole-body impedance vector components, resistance (*R*), reactance (*X*_*c*_), and phase angle (pA), were measured with a single-frequency 50 kHz analyzer STA-BIA (AKERN Bioresearch SRL, Pontassieve, Florence, Italy). Other parameters like Body Cell Mass (BCM: the protein rich compartment which is affected in catabolic states) and hydration status (total body (TBW), extracellular (ECW), and intracellular water (ICW)) were derived from electrical data. Measurements were obtained following standardized procedures [15]. The external calibration of the instrument was checked with a calibration circuit of known impedance value. Estimations of FFM and FM by BIA were obtained using gender-specific, BIA prediction equations recently developed by Sun et al. [16] in a large population that included extremes of BMI values. The fat mass index (FMI) was calculated through the normalisation of FM, obtained by the BIA, for height: FMI = FM (kg)/height (m)^2^. 

### 2.3. Biochemical Analyses

Subjects were instructed to fast over 12 hours and to refrain from any form of exercise for 48 hours, before blood collection. Female subjects were tested during the early follicular phase of their menstrual cycles (days 3–10). Fasting venous blood samples were drawn between 08.00 and 10.00 AM. Blood collection and handling were carried out under strictly standardized conditions, and clinical chemistry parameters were detected with dedicated commercial kits. In particular, total cholesterol, triacylglycerol, HDL- and LDL-cholesterol, free fatty acid (FFA), glycerol, glucose, insulin, C-reactive protein (CRP), plasma acylated and unacylated ghrelin, adiponectin, and leptin serum levels were measured. Leptin/adiponectin ratio (LAR) was calculated. Insulin resistance was evaluated using the Homeostasis Model Assessment (HOMA) [17] and Quantitative Insulin sensitivity Check Index (QUICKI) [18] using the following formulas:

 HOMA-IR = [(fasting insulin, *µ*U/mL) × (plasma glucose, mmol/L)]/22.5,
 QUICKI = 1/[log(glucose, mg/dL) + log(insulin, *µ*U/mL)].

### 2.4. Statistical Analysis

Data were described as mean and standard deviation (SD) if continuous and as percentage if categorical. 

We considered FM from DXA as the outcome variable and all the anthropometric, bioimpedance, and laboratory data as potential explicative variables. 

The predictive values of BMI and FM from BIA were compared to the FM from DEXA (overall predictive value, sensitivity, specificity, positive, and negative predictive values). Therefore, we considered the following cut-off values for the definition of obesity:

- FM ≥ 25% for men and ≥35% for women (at DXA and BIA) [3];
- BMI ≥ 30 kg/m^2^ [6].

The variance analysis and the Student *t*-test was used to assess the significance of differences in the averages; the *χ* ^2^ to compare the frequencies observed with those expected; Pearson's to evaluate the correlation existing between two continuous variables. 

Variables univariately proven to correlate with the outcome variable were entered a pool of potential contributors in multiple regression analysis. 

We estimated models using a forward likelihood stepwise method (cut-off probability for entry: 0.05). With each added variable, the discriminant function was recalculated, and any variable that no longer met the significance level was removed from the equation (cut-off probability for removal: 0.1). 

Some variables with similar biological significance were excluded from the logistic analysis, in order to avoid the confounding effect of collinearity (verified with Pearson's *r*, *t*-test, or *χ* ^2^). The best fitting model was chosen according to the value of the correlation coefficients *R* ^2^ (comparing the explained variance of the model's predictions with the total variance of the data) and the adjusted *R* ^2^ (*R* ^2^ adj), considering a correction for inclusion of variables. 

We considered a significance level equal to a 5% probability of error. 

Data were analysed using the SPSS for Windows 10.0 (SPSS Inc. 1989–1999) and the Win Episcope 2.0 (Facultad de Veterinaria di Saragozza (E), Wageningen University (N), and University of Edinburgh (GB)) statistical software packages.

## 3. Results

One hundred and three overweight or obese subjects were included in the study: 74 women (aged 41.5 ± 10.2 years) and 29 men (aged 43.8 ± 8.1 years); baseline characteristics are summarized in Table 1.

BIA showed a good predictive value in classifying subjects as obese when compared to DXA (overall predictive value 85.3%, sensitivity 85%), while BMI exhibited a very bad performance (overall predictive value 48.5%, sensitivity 47.5%) (Table 2). 

The results of the univariate analysis considering the correlation between FM from DXA and all anthropometric, bioimpedance, and laboratory parameters (Table 3) showed that

1. a good correlation was found between FM from DXA and BMI (*r* = 0.74), AFA (*r* = 0.59), waist and hip circumferences (*r* = 0.75). Although it is slightly lower, a statistically significant correlation was also observed between FM from DXA and skinfold thicknesses, waist, arm, and calf circumferences;
2. a good correlation was present between FM from DXA and FM from BIA (*r* = 0.91) and FMI (*r* = 0.86);
3. a good correlation was shown between FM from DXA and CRP (*r* = 0.43) and leptin levels (*r* = 0.57), and leptin/adiponectin ratio (*r* = 0.42). Although it is slightly lower, a statistically significant correlation was observed between FM from DXA and insulinemia, HOMA, and QUICKI indexes.

The multivariate regression analysis was performed using only the independent variables significantly correlated with the outcome variable in the univariate analysis: BMI, AFA, H, and FM from BIA, FMI, CRP, leptin, and LAR.

In the block model of the regression analysis, all the selected variables were included and *R* ^2^ and *R* ^2^  adj of the model were, respectively, 0.88 and 0.87. The strength of association between fat mass from DXA and independent variables was, in descending order, greater for FM from BIA (*r* = 0.91; *r* = 0.75), BMI (*r* = 0.73), AFA (*r* = 0.59), leptin (*r* = 0.57), FMI (*r* = 0.42), CRP (*r* = 0.42), and LAR (*r* = 0.41).

With the forward stepwise, three variables (FMI, CRP, and LAR) were omitted; *R* ^2^, *R* ^2^ adj, and the strength of association between fat mass from DXA and independent variables remained unchanged. 

When BMI entered the regression equation (at the third step), it accounted for 1.6% to the variance of the model (sig *F* change = 0.001).

Different models considering alternatively BMI or FM from BIA together with leptin and CRP levels were verified. The model including FM from BIA showed a better correlation (greater *R* ^2^ and *R* ^2^ adj) than the model using BMI (Tables 4 and 5). The inclusion in the model of the FMI instead of the FM from BIA did not result in any improvement of the model. 

The results and models identified have maintained their substantial validity for both genders and for different classes of BMI (less than or greater than 30 kg/m^2^) or age (less than or greater than 30 years) (data not shown).

## 4. Discussion

The results of the study showed that the BMI did not represent the main predictor of FM from DXA. FM from BIA and hip circumference showed a better association with FM from DXA than BMI. Moreover, models omitting BMI explained a greater part of the variance. These data were confirmed by the predictive value analysis where BMI showed a performance similar to a “coin flip.”

The boundary between health and disease in malnutrition (over- and undernutrition) in terms of body composition is crucial to accurately define criteria for intervention, and in particular methods and intensity of nutritional intervention, but it still represents a clinical challenge to be addressed.

The BMI formula was developed nearly 200 years ago by Adolphe Quételet. The index appeared for the first time in an article published on Proceedings of the Academy of Sciences [7] titled “*Recherches sur le poids de l'homme aux différens âges*” in 1833. A. Quételet devised the equation in 1832 in his quest to define the “normal man” taking into account a number of aspects, from his average arm strength to the age at which he marries. The equation was used to describe the standard proportions of the human build—the ratio between body weight and height in the average adult. Using data collected from several hundred countrymen, he found that body weight varied not in direct proportion to height but in proportion to the square of height (people 10% taller than average tended to be about 21% heavier). It is therefore not a measurement of adiposity, but merely an imprecise mathematical estimation, as shown in many papers [19–23].

Even if BMI represents an important epidemiological tool, as evidenced by the study by Ancel Keys [8], when considering the single individual, it cannot be considered a reliable diagnostic tool to define the degree of obesity that is necessary to define the intensity of the clinical interventions (nutritional, psychological, rehabilitation, surgical, and pharmacological interventions) that can be applied to the overweight or obese patient. The reason is that the BMI is not able to accurately assess the body composition, especially in terms of FM, FFM, and water content, whereas it is useful in defining the severity of obesity. It means that the predictive ability of BMI to identify obese subjects (FM > 25% for men and >35% for women) [3] is very poor, as shown in our study. In addition, the BMI is not able to discriminate two broad categories of subjects that deserve special attention in their therapeutic and rehabilitative pathway: patients suffering from “sarcopenic obesity”, who have a more marked disability, due to their reduced FFM, and the “normal weight-obese” subjects whose FM is increased despite a normal BMI, having a higher risk of comorbidities such as hyperlipidemia, coronary artery disease, hypertension, and diabetes [24]. Furthermore, change in BMI predicts neither change in FM nor in FFM, as demonstrated in different categories of patients [25, 26]. Finally, different studies show that BMI/FM relation is curvilinear especially at higher BMIs with a different association at different levels of BMI [27]. 

BMI significantly underestimates prevalence of obesity when compared to DXA direct measurement of body fat percentage. In our study, the predictive capacity of BMI to correctly classify subjects as obese is very low. In our sample, despite considering only overweight or obese subjects, BMI explained just 74% of the variance of FM. In other studies, based on the general population, the results are even worst [28].

On the other hand, despite representing the reference method in clinical practice to define body composition, DXA presents some critical aspects related to the fact that it is not available in all facilities treating obese subjects, to the necessity to use the same DXA device and analysis software for longitudinal evaluation and studies, and to its structural limitations that do not allow, in most cases, an effective assessment in particular of patients with a BMI > 40 mg/m^2^ [29]. 

Hence, we still need to measure and estimate body composition, reflecting nutritional intakes, losses, and expenses over time. Therefore, practical tools for this purpose and clinically useful biomarkers remain to be identified, in order to better characterize obese subjects and target their therapeutic and rehabilitative approaches.

In contrast with body weight and BMI, techniques for body composition measurement allow the measurement of tissue losses, by analyzing separately FFM and FM [30].

Moreover, different authors suggest that FFM and FM should be better normalized for body height (FFMI = FFM (kg)/height (m)^2^); FMI = FM (kg)/height (m)^2^, similarly to normalization of body weight for height through BMI calculation, to express the results of body composition [28, 31, 32]. In our study, this normalization did not improve the predictive value of the multivariate regression models. 

The validity of anthropometric variables and bioimpedance analysis has been questioned [33–35]. If anthropometry has not indifferent limits linked to the principles and clinical applications of BIA have been described in different studies since many years and reviewed in two position papers of the ESPEN [36, 37]. BIA is based on the ability of hydrated tissues to conduct electricity. The measurement of total body impedance allows the estimation of total body water by assuming that hydration status is constant. From total body water (60% is the proportion of body weight attributable to water in healthy adult), assuming that in muscle there is about 73% of water, and using validated equations and reference values, it is possible to estimate FFM and, by the difference between body weight and FFM, body fat, indirectly [38, 39]. Because of its simplicity, noninvasiveness, low-cost, quickness of use at bedside, and high interoperator reproducibility, BIA has emerged as the technique of choice for the systematic and repeated evaluation of FFM (and FM) in clinical practice [40]. 

As already stated by Deurenberg, however, several factors limit the valid application of BIA in the severely obese state: the assumption of a constant hydration status, body geometry, and body water distribution [41]. 

Different attempts were made to improve the predictive capacity of anthropometric parameters and bioimpedance analysis implementing their results with different biochemistry parameters (leptin, adiponectin, insulin levels, etc.) [20, 42]. In our study, models considering FM from BIA together with leptin concentrations seem to be better correlated with FM from DXA.

In other studies, the use of leptin levels improved the precision of BMI adjustment, whereas, as verified in our study, adiponectin to leptin/adiponectin ratio, insulin, and ghrelin levels did not. This effect was attributed to hyperleptinemia among obese subjects (in particular in women) [20]. It was previously suggested to incorporate leptin adjustments into a more accurate diagnosis of obesity considering also that a significant decrease of leptin affects long-term weight control [43, 44]. Moreover, increased leptin levels are associated with the inflammatory process and potentially the entire increased morbidity of obesity [45, 46]. 

Our study had several limitations. First of all, it was a cross-sectional study; longitudinal data would allow the quantification of outcomes related to adiposity in particular in “normal” BMI population and in sarcopenic obese subjects. Furthermore, our study was based on a convenience sample of small size considering only subjects with BMI between 25 and 40 kg/m^2^ and aged between 18 and 57 years. Therefore, the results that we found must be verified in other age classes and for BMI groups below and above this range.

Finally, an accurate definition of fat mass is necessary as one pivotal criterion for clinical interventions, in particular in tailoring nutritional intervention and to document its effectiveness. Considering that the BMI cannot be a reliable predictor of FM, we can hypothesize that the use of BIA in combination with other biomarkers (leptin levels in particular) could be very useful in defining the clinical features of the obese patient in order to better address the therapeutic and rehabilitative approaches, as long as the cost/effectiveness of DXA will not be favorable. 

## Figures and Tables

**Table 1:** Body composition and biochemical parameters of subjects studied^1^.

	F	M
*N*	74	29
Age (y)	41.6 ± 10.2	43.8 ± 8.1
DXA		
Fat tissue (kg)	35.0 ± 6.2	31 ± 7.2*
Android fat (%)	51.5 ± 5.2	45.1 ± 5.2*
Gynoid fat (%)	51.5 ± 5.0	34.6 ± 6.1*
Android/gynoid ratio	1.0 ± 0.1	1.3 ± 0.1*
Lean tissue (kg)	40.5 ± 5.4	59.3 ± 5.3*
Anthropometry		
BMI (kg/m^2^)	29.9 ± 3.2	31.0 ± 3.2
Waist (cm)	95.6 ± 9.3	104.6 ± 7.2*
Hips (cm)	109.1 ± 7.1	106.0 ± 6.3
WHR	0.88 ± 0.07	0.98 ± 0.03*
TSF (mm)	28.8 ± 7.1	17.4 ± 5*
BSF (mm)	19.2 ± 8.1	9.7 ± 5.2*
SISF (mm)	31.0 ± 9.2	27.8 ± 10.1
SSSF (mm)	31.9 ± 8.2	30.8 ± 9.4
AFA (cm^2^)	5.2 ± 1.0	4.9 ± 1.1
AC (cm)	33.2 ± 3.1	34.2 ± 2.3
CC (cm)	38.6 ± 2.2	39.7 ± 2.2*
AMA (cm^2^)	40.6 ± 11.2	56.2 ± 10.2*
MAC (cm)	24.2 ± 2.3	28.7 ± 2.2*
Bioimpedance analysis		
*R* (Ω)	554.7 ± 58.1	454.6 ± 50.1*
*X*_*c*_(Ω)	57.1 ± 7.1	54.4 ± 7.3
pA (°)	5.9 ± 0.7	6.8 ± 0.9*
TBW (L)	35.5 ± 3	49.2 ± 4.2*
ECW (L)	16.4 ± 1.1	20.8 ± 2.2*
ICW (L)	19.1 ± 2.2	28.4 ± 3.3*
FM-BIA (kg)	30.5 ± 6.2	26.6 ± 6.3*
FMI	11.6 ± 2.1	8.8 ± 2.4*
FFM-BIA (kg)	48.3 ± 4.2	67.3 ± 5.3*
BCM (kg)	24.5 ± 4.2	34.4 ± 7.1*
Laboratory parameters		
CT (mg/dL)	200.3 ± 38.2	212.3 ± 35.1*
HDL cholesterol (mg/dL)	56.4 ± 12.1	43 ± 8.1
LDL cholesterol (mg/dL)	125.0 ± 30.1	137.3 ± 33.1
CT/HDL ratio	3.6 ±0.71	5.1 ± 1.3*
TG (mg/dL)	94.4 ± 42.2	160.0 ± 103.3*
Free fatty acids (mM/L)	0.42 ± 0.2	0.39 ± 0.1
Glycerol (mM/L)	0.14 ± 0.04	0.14 ± 0.04
Glycaemia (mg/dL)	88.6 ± 9.2	93.5 ± 9.0*
Insulin (IU/mL)	9.4 ± 4.2	11.7 ± 5.3*
HOMA	2.1 ± 1.2	2.7 ± 1.0*
QUICKI	0.35 ± 0.02	0.34 ± 0.02*
CRP (mg/L)	0.4 ± 0.6	0.3 ± 0.4
Adiponectin (ng/mL)	93.9 ± 52.1	57.4 ± 28.2*
Leptin (pg/mL)	272.7 ± 163.3	80.3 ± 51.3*
Leptin/adiponectin ratio	3.8 ± 3.4	2.3 ± 3.2*
Ghrelin (pg/mL)	442.4 ± 260.4	240.3 ± 131.3*

^1^Values are means ± SD.

*P < 0.05.

Legend: BMI: body mass index; WHR: waist hip ratio; BSF: biceps skinfold thickness; TSF: triceps skinfold thickness; SISF: suprailiac skinfold thickness; SSSF: subscapular skinfold thickness; AC: arm circumference; CC: calf circumference; AMA: arm muscle area; AFA: arm fat area; MAC: muscle arm circumference; *R*: resistance; *X*_*c*_: reactance; pA: phase angle; TBW: total body water; ECW: extracellular water; ICW: intracellular water; FM-BIA: fat mass estimate through bioimpedance analysis; FMI: fat mass index; FFM-BIA: fat free mass estimate through bioimpedance analysis; BCM: body cell mass; CT: cholesterol total; TG: triglycerides; HOMA: homeostasis model assessment; QUICKI: quantitative insulin sensitivity check index; CRP: C-reactive protein.

**Table 2:** Predictive value of BIA and BMI towards DXA.

		DXA		
		Nonobese (FM < 25% M, < 35% F)	Obese (FM ≥ 25% M, ≥ 35% F)	
BMI	<30 kg/m^2^ ≥30 kg/m^2^	2 0	53 48	Overall predictive value: 48.5% Sensitivity: 47.5% Specificity: 100% Positive predictive value: 100% Negative predictive value: 3.6%

BIA	Non-obese (FM < 25% M, 35% F) Obese (FM ≥ 25% M, 35% F)	2 0	15 85	Overall predictive value: 85.3% Sensitivity: 85% Specificity: 100% Positive predictive value: 100% Negative predictive value: 11.8%

**Table 3:** Univariate analysis: correlation between FM from DXA and anthropometry, bioimpedance, and laboratory parameters.

	*r*
Anthropometry	
BMI (kg/m^2^)	0.74*
Waist (cm)	0.4*
Hips (cm)	0.75*
WHR	0.16
TSF (mm)	0.52*
BSF (mm)	0.5*
SISF (mm)	0.48*
SSSF (mm)	0.32*
AFA (cm^2^)	0.59*
AC (cm)	0.51*
CC (cm)	0.5*
AMA (cm^2^)	0.06
MAC (cm)	0.04
Bioimpedance analysis	
*R* (Ω)	0.05
*X*_*c*_ (Ω)	0.14
pA (°)	0.2*
TBW (L)	0.06
ECW (L)	0.07
ICW (L)	0.12
FM-BIA (kg)	0.91*
FMI	0.86*
FFM-BIA (kg)	0.06
BCM (kg)	0.2*
Laboratory parameters	
CT (mg/dL)	0.08
HDL cholesterol (mg/dL)	0.07
LDL cholesterol (mg/dL)	0.08
CT/HDL ratio	0.19
TG (mg/dL)	0.09
Free fatty acids (mM/L)	0.03
Glycerol (mM/L)	0.02
Glycaemia (mg/dL)	0.1
Insulin (IU/mL)	0.24*
HOMA	0.2*
QUICKI	0.18
CRP (mg/L)	0.43*
Adiponectin (ng/mL)	0.1
Leptin (pg/mL)	0.57*
Leptin/adiponectin ratio	0.42*
Ghrelin (pg/mL)	0.2

*P < 0.05.

Legend: BMI: body mass index; WHR: waist hip ratio; BSF: biceps skinfold thickness; TSF: triceps skinfold thickness; SISF: suprailiac skinfold thickness; SSSF: subscapular skinfold thickness; AC: arm circumference; CC: calf circumference; AMA: arm muscle area; AFA: arm fat area; MAC: muscle arm circumference; *R*: resistance; *X*_*c*_: reactance; pA: phase angle; TBW: total body water; ECW: extracellular water; ICW: intracellular water; FM-BIA: fat mass estimate through bioimpedance analysis; FMI: fat mass index; FFM-BIA: fat free mass estimate through bioimpedance analysis; BCM: body cell mass; CT: cholesterol total; TG: triglycerides; HOMA: homeostasis model assessment; QUICKI: quantitative insulin sensitivity check index; CRP: C-reactive protein.

**Table 4:** Fat mass prediction (FM from DXA)—multivariate regression analysis.

		Variables in the model	*B*	Sig. *t* changes	*R* ^2^	*R* ^2^ adj
		Constant	−10.55	0.032		
		BMI	0.285	0.042		
		AFA	0.628	0.034		
		H	0.117	0.028		
	Block model	FM-BIA	0.739	0.000	0.879	0.868
		FMI	0.288	0.345		
		CRP	0.0036	0.995		
Model 1		Leptin	0.00714	0.01		
		LAR	0.0594	0.599		
		Constant	−11.021	0.02		
		FM-BIA	0.645	0.000		
	Forward stepwise selection	Leptin	0.00545	0.004	0.877	0.871
		BMI	0.245	0.058		
		H	0.131	0.11		
		AFA	0.627	0.32		

		Constant	6.818	0.000		
	Block model	FM-BIA	0.868	0.000	0.852	0.847
		Leptin	0.00629	0.002		
Model 2		CRP	0.477	0.41		
		Constant	6.642	0.000		
	Forward stepwise selection	FM-BIA	0.876	0.000	0.851	0.848
		Leptin	0.00681	0.001		

		Constant	−10.21	0.011		
	Block model	BMI	1.338	0.000	0.68	0.67
		Leptin	0.0153	0.000		
Model 3		CRP	0.842	0.321		
		Constant	−11.025	0.005		
	Forward stepwise selection	BMI	1.369	0.000	0.677	0.67
		Leptin	0.01638	0.000		

Legend: BMI: body mass index; AFA: arm fat area; H: hips circumference; FM-BIA: fat mass estimate through bioimpedance analysis; CRP: C-reactive protein; LAR: leptin-adiponectin ratio.

**Table 5:** Forward stepwise multivariate regression analysis (FM from DXA as dependent variable).

	Step	Variables entered	*R* ^2^	*R* ^2^ change	Sig. *F* change	*R* ^2^ adj
	1	Constant FM-BIA	0.828		0.000	0.826
	2	Leptin	0.847	0.02	0.001	0.844
Model 1	3	BMI	0.863	0.016	0.001	0.859
	4	H	0.871	0.008	0.019	0.866
	5	AFA	0.877	0.006	0.032	0.871

Model 2	1	Constant FM-BIA	0.831		0.000	0.829
	2	Leptin	0.851	0.019	0.001	0.848

Model 3	1	Constant BMI	0.541		0.000	0.537
	2	Leptin	0.677	0.135	0.000	0.67

Legend: BMI: body mass index; AFA: arm fat area; H: hips circumference; FM-BIA: fat mass estimate through bioimpedance analysis.
